# Evaluation of Water Provision Ecosystem Services Associated with Land Use/Cover and Climate Variability in the Winike Watershed, Omo Gibe Basin of Ethiopia

**DOI:** 10.1007/s00267-021-01573-9

**Published:** 2021-12-08

**Authors:** Abreham Berta Aneseyee, Teshome Soromessa, Eyasu Elias, Tomasz Noszczyk, Gudina Legese Feyisa

**Affiliations:** 1 Department of Natural Resource Management, Wolkite University, Ethiopia, Wolkite, Ethiopia; 2 Center of Environmental Science, College of Natural and Computational Sciences, Addis Ababa University, Addis Ababa, Ethiopia; 3 Department of Land Management and Landscape Architecture, Faculty of Environmental Engineering and Land Surveying, University of Agriculture in Krakow, Krakow, Poland

**Keywords:** InVEST model, Land-use change, Spatiotemporal changes, Water yield, Winike watershed

## Abstract

The provision of freshwater is essential for sustaining human life. Understanding the water provision modelling associated with the Land Use/Cover (LUC) change and climatic factors is vital for landscape water resource management. The Winike watershed is the largest tributary in the upper Omo Gibe basin of Ethiopia. This research aims to analyze the spatial and temporal change in the water yield to investigate the water yield contribution from the watershed based on the variation in input parameters. The Integrated Valuation of Ecosystem Services and Tradeoffs Tool (InVEST) water yield model was used to evaluate the spatial and temporal variation of the water yield in different years (1988, 1998, 2008 and 2018). The data required for this model include LUC data from satellite images, reference evapotranspiration, root depth, plant available water, precipitation, season factor (*Z*), and a biophysical table. The analysis of LUC change shows a rapid conversion of grazing land, shrubland, and forest land into cultivated land. There has been a significant variation in water provision, which increased from 1.83 × 10^9^ m^3^ in 1988 to 3.35 × 10^9^ m^3^ in 2018. Sub-watersheds 31, 32, and 39 in the eastern part of the watershed contributed more water due to higher precipitation and lower reference evapotranspiration. The major increase in the contribution of water yield was in built-up land by 207.4%, followed by bare land, 148.54%, and forest land by 63%. Precipitation had a greater impact on water yield estimation compared with the other input parameters. Hence, this research helps decision-makers to make informed decisions regarding new policies for LUC change improvement to maintain the water resources in the Winike watershed.

## Highlights

- Spatiotemporal changes in water yield from 1988 to 2018 were investigated.
- The water yield of each LUC was analyzed using the InVEST model.
- The main driver of increased water yield is climate factors.

## Introduction

The world population depends on water resources for sustaining its life and for economic purposes (Scordo et al. 2018). However, less than 1% of freshwater is accessible to the global community. Its uneven spatial and temporal distribution, along with overexploitation of water resources by humans, which causes scarcity of water (Mekonnen and Hoekstra 2011), further exacerbate the problem. Anthropogenic activities are major causes of the scarcity of freshwater (Murphy and Kapelle 2014; Szewrański et al. 2018). Economic growth together with population expansion cause land use to change significantly, which leads to further degradation of water resources (Assessment 2005; Sharma et al. 2020). This is because human livelihood and economic growth depend on ecosystem services such as food, water, and energy (Casagrande et al. 2021; Sahle et al. 2019). This, in turn, might jeopardize water sustainability due to anthropogenic and natural factors. Climate change has also become a threat to global human populations (Bangash et al. 2013; Gain and Wada 2014).

Water yield is the main regulatory ecosystem service, which contributes to the wellbeing of people, ensuring the development of irrigation, and improvement in the standard of living (Cudennec et al. 2007). Water yield is defined as the maintenance of water by ecosystems within a certain period (Xu et al. 2016). Other authors also defined water yield as the sum of surface runoff from the landscape (Chiang et al. 2019; Tallis et al. 2011). The relative amount of water in a given landscape affects the quality of the environment by either increasing or decreasing land productivity (Shoyama and Yamagata 2014; Srichaichana et al. 2019). Understanding the hydrological processes affected by the land-use change is essential for sustainable water resources management (Narsimlu et al. 2013; Walega and Salata 2019) because LUC changes affect water yield by interrupting the hydrological processes within the landscape (Aghsaei et al. 2020; Assessment 2005; Gebremicael et al. 2013; Gwate et al. 2015). Some examples of the effects that LUC change has been decreased streamflow due to the growth of agricultural land causing water withdrawal for irrigation and urban consumption (Bian et al. 2017); surface runoff reduction by 22 mm over 20 years due to the upstream land-use change (increase in forest land and grassland areas) in the Loess Plateau of China (Yan et al. 2018; reduced Alento River Catchment (UARC, southern Italy)) water yield and its actual evapotranspiration increased due to afforestation (Nasta et al. 2017); maximized runoff and minimized recharge of groundwater after grassland was replaced by agriculture and bare land in the Zanjan Rood catchment, Iran (Ghaffari et al. 2010); and progressive urban development affecting the hydrologic cycle in the Prądnik River basin, Poland (Lepeška et al. 2020). Moreover, findings by Lotz et al. (2018) in China also showed that the conversion of agricultural land into forest decreased water yield. However, vegetation type, precipitation intensity, soil permeability, topography, and geomorphology are complex factors that affect water yield assessment as well (Zhang et al. 2012).

National resource management policies, rapid socioeconomic growth, and climate instability are key factors leading to LUC change (Dwarakish and Ganasri 2015). Particularly in areas where the availability of water is too low, alterations in LUC result in water scarcity and water quality deterioration. Therefore, evaluating the effect of the LUC and climate change on water yield is vital for the sustainable management of a river basin (Ahiablame and Shakya 2016).

Reliable modelling of watershed processes is a critical part of aiding decision-makers and managers to sustain and improve ecosystem services (such as recreational opportunities, drinking water supply, and energy production) (Schröter et al. 2005). Various hydrologic models (Bieger et al. 2015) and land-cover data sources (Walega and Salata 2019) were used to assess the impacts of LUC change on the hydrologic response in a landscape. Among these models, the Integrated Valuation of Ecosystem Service and Trade-offs (InVEST) water yield model has been commonly applied to assess water provision associated with LUC change within a landscape. It can be useful for supporting decision-making (Arunyawat and Shrestha 2016) and has been widely used for a variety of research and planning applications (Bai et al. 2013; Bangash et al. 2013; Boithias et al. 2014). Besides, mapping and quantifying of water provision is used to avoid unintended impacts on the provision and production of services (Bastola et al. 2019).

The InVEST water yield model has easily operated software, minimum data needs, and computer requirements. Still, it offers more explicit output data for an annual timeframe and is suitable for areas with inadequate data coverage (Ibrahim et al. 2015; Komi et al. 2017; Vogl et al. 2016). It is a widely-used open-source tool available for free and appropriate for ecosystem service modelling (Redhead et al. 2016). The quick water yield generation for large geographical areas and spatially explicit nature of the model could facilitate the decision-making process by demonstrating the degradation of water resources. This could be achieved by identifying hotspots potentially in need of a vital intervention in land management and increased monitoring of hydrological ecosystem services at minimum costs (Dimobe et al. 2015; Lüke and Hack 2018; Vogl et al. 2016). These characteristics made the model attractive to use as compared to some complex hydrological models such as SWAT (Soil and Water Assessment Tool).

In the Omo Gibe basin of Ethiopia, four dams (I, II, III, and IV) are constructed and fifth and sixth were proposed, which would significantly contribute to the green economy of Ethiopia by generating 2800 MW of power (Aneseyee et al. 2019). This is possible because the basin has potential sources of water and many tributaries. However, human factors such as sedimentation, deforestation, agricultural exploitation, invasive species, urbanization, and pollution coupled with climate change have become a threat and harmed important ecosystem services such as water yield (Aneseyee et al. 2020). Some hydrological modelling research projects focusing on SWAT have been undertaken for the Omo Gibe basin; such as those by Choto and Fetene (2019), Estifanos and Gebremariam (2019), Takalaa and Tamamc (2016) and Chaemiso et al. (2016). However, the majority of previous research attempts on the availability of water in the Omo Gibe basin was limited (Chaemiso et al. 2016). Some of the gaps in the current knowledge can be summarized as follows: (1) While the ecosystem services assessment has made significant progress in recent decades, existing methods largely focus on estimating the total output from ecosystems (Martín-López et al. 2014; Yang et al. 2020a), which cannot reflect the ecosystems in full spectrum; they cannot distinguish ecosystems with the same assessment results but of various sizes (Shi et al. 2021). (2) In terms of a temporal scale, few studies followed a long, continuous timeline (over 20 years). Most existing research is limited to a short period (Shi et al. 2020; Xie et al. 2017). (3) Even though numerous studies target ecosystem services, quantitative relationships between influencing factors and ecosystem services have not been pinpointed (Ren et al. 2020).

Therefore, compared with previous studies, the objectives of this paper are (1) to analyze the spatial and temporal changes in water yield over the last 30 years; (2) to investigate the contribution of the watershed’s water yield to the main river for hydropower production; (3) to compare the impact of LUC change and climate variability on water yield, and 4) to prioritize the sub-watershed’s water yield and validate the model’s performance.

The authors decided to tackle the evaluation of water provision ecosystem services in their study because although similar investigations have often been the core issue of research (Shi et al. 2021; Stosch et al. 2017; Vardon et al. 2019), they have been relatively rare in Ethiopia and the north-eastern part of Africa. What is more, the land use/cover and climate data are broadly applied and help identify anthropogenic changes in space and watersheds.

The authors believe the study offers universal values and a valuable contribution of new knowledge of the evaluation of water provision ecosystem services from the international point of view. The novelty of this research is the field assessments and laboratory analysis supported by GIS and remote sensing, resulting in water yield potential (temporal and spatial). The InVEST water yield model used in the study is a foundation for further comparative analyses based on appropriate expert opinions. It may be applied to other areas where data on land use/cover and climate are available. The spatial evaluation of water resources at the watershed level under LUC and climate change scenarios can help identify vulnerable locations for adequate adaptation, planning, and implementation of responses. Therefore, this study is innovative and important for mapping water yield, water consumption, and water supply.

## Materials and Methods

### Study Area

**Fig. 1:**
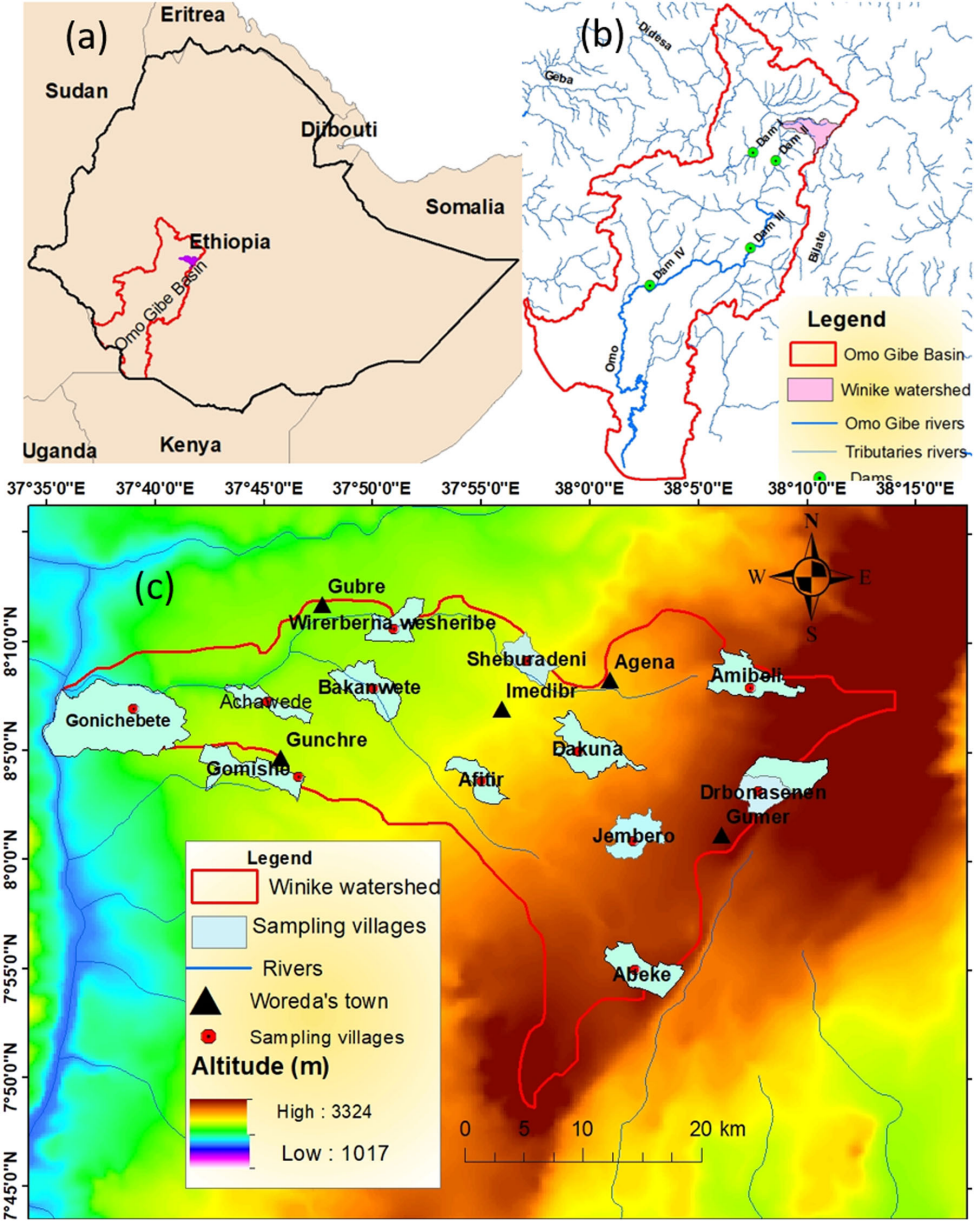
Map of the watershed. Watershed in Ethiopia (**a**) and hydropower dams (**b**) showing the sub-watersheds (**c**) (sampling villages are used to collect the local Ecosystem services valuation data)

The study site is located in the regions of the Southern Nations, Nationalities, and Peoples’ Region state (SNNPR) of Ethiopia within the basin of Omo Gibe with coordinate bounds of 7°40’N to 8°20’N and 37°40’E to 38°10’E and a total area of 1091.8 km^2^ (Fig. 1). The Winike River is one of the tributaries of the Omo Gibe basin situated in five districts of the Guraghe zone and one district of the Silte zone.

**Fig. 2:**
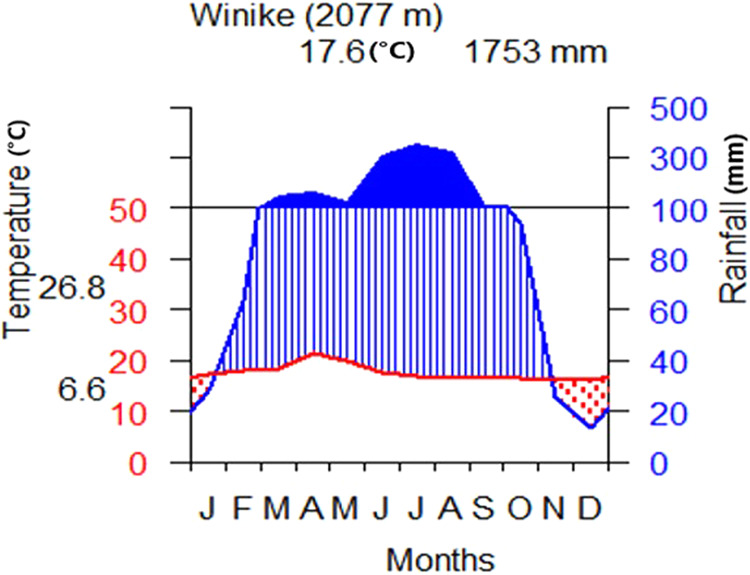
Climatogram for the watershed (the climate data analysis based on data from 1988 to 2018)

Its lowest altitude is 1022 m ASL at the Gibe gorge and the highest altitude, 3324 m ASL in the Bozebar area of the watershed. Its slope gradients vary from zero to 89.9°. The annual rainfall varies from 856 mm to 1600 mm with a bimodal distribution with a mean annual total of 1753 mm (Fig. 2). June to September is the main rain season (summer), and March to April is the short rain season, which is spring. This season can provide rain that is sufficient for farming. The maximum and minimum mean temperature values are 26.8 and 6.6 °C, respectively, with a mean temperature of 17.7 °C. The upper part of the watershed is dominated by Eucalyptus plantations, whereas the lower part is occupied by Acacia vegetation (*Acacia polyacantha*) and grassland.

### The InVEST Water Yield Model

The InVEST water yield model (Hydropower/Water Yield, InVEST v3.6.0) was used to analyze the water yield in the watershed. It also demonstrated the watershed contribution to hydropower production (Sharp et al. 2018) and the downstream ecosystems. The InVEST water yield model input data were pre-processed in ArcGIS to normalize pixel size before using them in the model (Table 1).

**Table 1:** Data required for the InVEST water yield model

Categories of data	Types	Sources	Range of sensitivity analysis
1 LUC^a^	Raster	United State of Geological Survey	n/a
2 Temperature data^a^	Numeric	National Mereology Agency	n/a
3 Watershed	Vector	EthioGIS	n/a
4 Sub-watersheds	Vector	Digital elevation model	n/a
5 Root depth	Raster	Yang et al. (2016)	±10
6 ET_0_^a^	Raster	Hargreaves and Allen (2003)	±10
7 Precipitation^a^	Raster	National Mereology Agency	±10
8 Plant available water content	Raster	Laboratory analysis	±10
9 Consumptive water	Numeric	Field survey	n/a
10 Z	Constant	Sharp et al. (2018)	±10
11 Kc	Numeric	Allen et al. (1998)	±10

^a^ Average data used for the years (1988, 1998, 2008, and 2018)

The total annual water yield (Y) in the study watershed was estimated by the annual rainfall (P) minus the actual annual evapotranspiration (AET) (Eq. 1). In other words, the difference between all water falling as precipitation over the watershed and evapotranspiration loss from the watershed. We used Eq. (1) by Budyko et al. (1974) to calculate the annual water provision (Y(x)) for a pixel of the landscape (x).

1 $$Y\left( x \right) = \left( {1 - \frac{{AET\left( x \right)}}{{P\left( x \right)}}} \right)P\left( x \right)$$

where AET(x) – the actual annual evapotranspiration in pixel x and P (x) – the annual precipitation in pixel x.

For vegetative LUC, the evapotranspiration portion of the water balance $\left( {\frac{{{{{{{\mathrm{AET}}}}}}\left( {{{{{\mathrm{x}}}}}} \right)}}{{{{{{{\mathrm{P}}}}}}\left( {{{{{\mathrm{x}}}}}} \right)}}} \right)$ is analyzed based on the Budyko curve supported by Fu (1981) and Zhang et al. (2004) given in Eq. (2).

2 $$\frac{{{{{{{\mathrm{AET}}}}}}\left( {{{{{\mathrm{x}}}}}} \right)}}{{{{{{{\mathrm{P}}}}}}\left( {{{{{\mathrm{x}}}}}} \right)}} = 1 + \frac{{{{{{{\mathrm{PET}}}}}}\left( {{{{{\mathrm{x}}}}}} \right)}}{{{{{{{\mathrm{P}}}}}}\left( {{{{{\mathrm{x}}}}}} \right)}} - \left[ {1 + \left( {\frac{{{{{{{\mathrm{PET}}}}}}\left( {{{{{\mathrm{x}}}}}} \right)}}{{{{{{{\mathrm{P}}}}}}\left( {{{{{\mathrm{x}}}}}} \right)}}} \right)} \right]^{\omega \ast 1/\omega }$$

where PET(x) – the potential evapotranspiration and ω(x) – a non-physical parameter that shows the natural properties of the soil climate zone.

Potential evapotranspiration (x) is calculated with Eq. (3)

3 $${{{{{\mathrm{PET}}}}}}\left( {{{{{\mathrm{x}}}}}} \right) = {{{{{\mathrm{Kc}}}}}}\left( {{{{{\mathrm{x}}}}}} \right) \times {{{{{\mathrm{ETo}}}}}}\left( {{{{{\mathrm{x}}}}}} \right)$$

where Kc(x) – the coefficient of LUC evapotranspiration used to adjust for reference evapotranspiration (the value is provided in Table S1) and *ET*_0_(x) – the annual reference evapotranspiration per pixel x.

The non-physical parameter, ω(x), proposed by Donohue et al. (2012) for the InVEST model, is calculated with Eq. (4)

4 $$\omega \left( {{{{{\mathrm{x}}}}}} \right) = {{{{{\mathrm{Z}}}}}}\frac{{{{{{{\mathrm{PAWC}}}}}}\left( {{{{{\mathrm{x}}}}}} \right)}}{{{{{{{\mathrm{P}}}}}}\left( {{{{{\mathrm{x}}}}}} \right)}} + 1.25$$

where Z – the season factor and PAWC – Plant Available Water Content

#### Input data pre-processing for the model

All the required input datasets for the InVEST water yield model were (Table 1) projected using the Universal Transverse Mercator (UTM) of WGS84 zone 37°N and be resampled with a spatial resolution of 30 m. The images were terrain-corrected projected to the Universal Transverse Mercator (UTM).

##### GIS and land use/cover analysis

Landsat satellite images for the years 1988 (Landsat 5), 1998 (Landsat 5), 2008 (Landsat 7), and 2018 (Landsat 8) were obtained from the United States Geological Survey (USGS) data portal (https://earthexplorer.usgs.gov) to analyze the LUC. The selected images were taken in the dry season when the monthly cloud cover is the lowest. Due to the failure of the Scan Line Corrector (SLC) in 2003, the 2008 images acquired by the sensor exhibit data gaps (striping). Hence, we applied image gap-filling using a gap-filling tool in ENVI v5.3.

Radiometric calibration to reflectance value, geometric correction, and Quick Atmospheric A correction algorithm was applied to the images before they were classified. A supervised image classification method was employed using the Mahalanobis distance classification, and the Ground Reference Points (GRP) data were collected using a GPS receiver to convert a vector file. Then, a Region of Interest (ROI) was determined in ENVI v5.3 software. Using the ROI, the spectral signature of each LULC type has been extracted. Based on the analysis and gathered information, eight types of LUCs were categorized. An error matrix such as accuracy assessment was analyzed to indicate the reliability of the LUC classification and validation. The Kappa coefficient was also used to show the conformity of the classified image with the reference data.

##### Root depth

Rooting depth (cm) is the accessible soil profile for water storage. It is defined as the soil depth at which 90% of root biomass occurs. The study watershed root depth was determined based on carbon cost-benefit model developed by Guswa (2008) and Yang et al. (2016). The carbon cost-benefit model incorporates seven parameters; the root respiration, root length density, specific root length, photosynthetic water use efficiency, the fraction of growing season within a year, and mean annual transpiration rate for a given root depth.

##### Reference evapotranspiration (ET_0_)

The reference evapotranspiration was estimated based on the modified Hargreaves method (Hargreaves and Allen 2003) using Eq. (5). The Hargreaves method requires the parameter of precipitation, maximum and minimum average temperatures, and extraterrestrial radiation (RA) (mm/day). Thirty-year (30) data on rainfall and temperature were obtained for the watershed from the National Meteorology Agency (NMA) of Ethiopia. The RA was determined in the R software Package SPEI (Guo et al. 2016), by inputting the latitudinal location of the watershed (Table S2).

5 $$\begin{array}{ll}ET_0 = 0.0013 \times 0.408 \times {{{\mathrm{RA}}}} \times \left( {T_{mean} + 17} \right)\\ \qquad\quad \times \,\left( {TD - 0.0123 \times P} \right)^{0.76}\end{array}$$

where T_mean_ – the average daily temperature (average of the mean daily maximum and minimum temperature (°C)); RA – the extraterrestrial radiation (mm/day); ET_0_ – the reference evapotranspiration; and TD – the temperature ranges (°C).

**Fig. 3:**
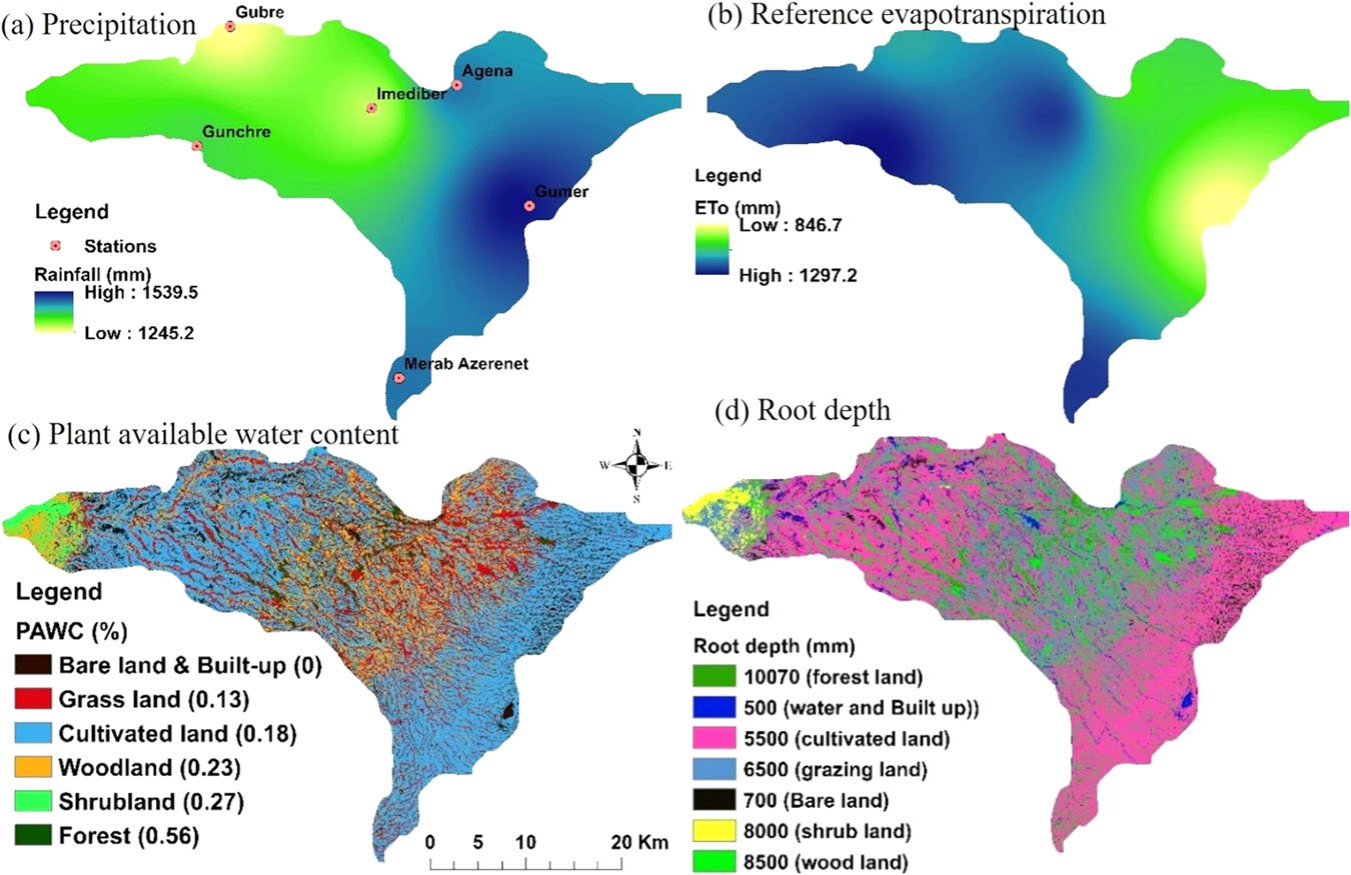
Input parameters for the InVEST water yield model. **a** Precipitation, **b** reference evapotranspiration, **c** plant available water content, and **d** root depth

The interpolation technique of IDW (inverse distance weighted) in ArcGIS v10.4 (ESRI, Inc., Redlands, CA, USA) was used to generate a raster spatial map for the mean annual ET_0_ (mm) and P (mm) values for the InVEST model input (Fig. 3a, b).

##### Plant available water content (PAWC)

Plant Available Water Content (PAWC) is the volumetric (mm) plant available water content, expressed as the rate of water that can be held in the soil for use by vegetation (Fig. 3c). The estimation of PAWC developed by Zhou et al. (2005), based on soil texture (sand%, silt%, and sand%) and Organic Matter (OM) content in the soil for each LUC type is calculated using Eq. 6.

6 $$\begin{array}{ll}PAWC = 54.509 - 0132 \times sand - 0.003 \times \left( {sand} \right)^2 \\ \qquad\qquad\quad- \,0.055 \times silt - 0.006 \times \left( {silt} \right)^2 - 0.738\\ \qquad\qquad\quad \times \,clay + 0.007 \times \,\left( {clay} \right)^2 - 2.688\\ \qquad\qquad\quad \times \,OM + 0.501 \times \left( {OM} \right)^2\end{array}$$

These soil parameters were analyzed in the Wolkite soil laboratory by taking 100 soil samples of representative land-use types.

##### Season factor (Z)

The season factor (*Z*) is estimated with three different methods. The first method is for calculating the *Z* parameter from an available ω parameter (Xu et al. 2013) using the rearranged Eq. (3) as ${{{{{\mathrm{Z}}}}}} = \frac{{\left( {\omega - 1.25} \right)P}}{{{{{{{\mathrm{PAWC}}}}}}}}$ and its value is 32. The second way to determine the season factor (Z) is to take 1/5 of the number of rain events (N) per year (0.2*N) (Donohue et al. 2012). The number of rainfall events (N) was determined with Ethiopian National Meteorology Agency (NMA) data, which indicates that the mean number of rainy days per year in the Winike watershed was approximately 145. Thus, the *Z* parameter value is 29. The third method is based on observed and calibrated streamflow data for the last 30 years, which resulted in the average *Z* value of 12. Of course, the Z parameters would be estimated in different sources. However, the second method was used to estimate explicitly the overall spatial and temporal water yield model since it best reflected the reality of the Ethiopian environment, but the first and the third were somehow complicated to get the precise data for Ethiopia. Moreover, the three values were used for sensitivity analysis of the water yield similarly to the sensitivity analysis for precipitation and evapotranspiration.

#### Water yield contribution by the watershed

According to Sharp et al. (2018), the actual amount of water from the watershed that reaches the dam reservoir (realized supply (d)) is calculated as a difference between the total water yield and consumptive water use (Eq. 7). For this analysis, we prepared a demand table with input for the InVEST model to show how much water is consumed by each land use/cover type. We investigated water consumed by agriculture and urban land. Therefore, each land-use type in the watershed either contributes to hydropower production or consumes water.

7 $$V_{in} = Y - U_d$$

where *V*_*in*_ – the realized supply (volume of water inflow to a reservoir from upstream of the watershed), *U*_*d*_ – the total anthropogenic water consumption in the watershed, and *Y* – the total water yield, calculated with the InVEST model.

We investigated water consumed by agriculture and urban land. Industrial withdrawals which are not returned to the water body are recognized in urban land. Three private water abstraction companies producing bottled drinking water are located in the Winike watershed. These water packing companies are Aden, Fiker, and Waw. Their approximate annual water withdrawal rate was determined by consulting each of the water company offices.

In urban land, consumptive use can be calculated as the product of population density and per capita consumptive use. Therefore, data on water consumed by individual households for their daily activities was collected through a HH survey by selecting twelve (12) villages (*kebeles*) from six districts using purposive sampling. A sample of households was investigated with the Cochran and Banner (1977) formula. Thus, human water consumption was estimated based on individual water usage (l/day/person) multiplied by the size of the population in the watershed.

Water used by livestock that is not returned to the reservoir must be considered for agricultural land. According to Sileshi et al. (2003), the annual water requirement for sheep and goats is 0.011 m^3^/day, cattle consumption is 0.045 m^3^/day, and consumption by horses, mules, camels, or assess is 0.045 m^3^/day. The population data for the livestock was obtained from the Guraghe Zone Department of Finance and Economic Development (GZDFED) (2016) of Ethiopia. The total water consumption by livestock was calculated from the number of livestock specimens multiplied by individual livestock water consumption value, according to Sileshi et al. (2003).

#### Model sensitivity

InVEST water yield model uncertainty originated from the uncertainty of climate data (i.e., the variability of precipitation and reference evapotranspiration) (McGlynn et al. 2012; McMahon et al. 2013; Sahle et al. 2019). Therefore, checking the credibility of the source data for precipitation and PET helps reduce the error in the modelled water yield.

The available climate data obtained from the different sources located over the watershed might have generated an error of ±10 for the water yield analysis (Hamel and Guswa 2015). This shows that the water yield result changes significantly for a 10% change in the value of parameters, and the model is highly sensitive to errors as regards estimating water yield. Therefore, applying uniformly ±10% to each water yield input model parameter in the baseline climate input data across the landscape explicitly shows efficient decision-making on regulating water provision (Hamel and Guswa 2015). Finally, the models were run independently using the InVEST model for each of these parameters’ variations to determine whether or not each parameter had a significant effect on water yield results.

#### Model validation

To verify the applicability and reliability of the model, we validated the exported InVEST water yield results against observed water yield available from Ethiopian Ministry of Water, Irrigation, and Energy (MOWIE) data (1988–2018). The output of the InVEST water yield model was provided in m^3^/year and the observed streamflow data was expressed in m^3^/s. For consistent analysis, the observed data had to be converted to m^3^/year based on the streamflow (m^3^/s) data from five gauging stations.

The Coefficient of Determination (R^2^), Residual Root Mean Square (RRMSE), Nash-Sutcliffe Efficiency (NSE), and average Percentage Bias Error (PBIAS) (Eqs. 8 to 11) were used to validate the performance of the model (Gyamfi et al. 2016; Munoth and Goyal 2019). This was necessary to determine the applicability of the InVEST water yield model for the watershed.

8 $${{{{{\mathrm{RRMSE}}}}}} = \frac{{\sqrt {\frac{1}{{{{{{\mathrm{n}}}}}}}} \mathop {\sum}\nolimits_{j = 1}^n {\left( {{{{{{\mathrm{P}}}}}}_{{{{{\mathrm{i}}}}}} - {{{{{\mathrm{O}}}}}}_{{{{{\mathrm{i}}}}}}} \right)^2} }}{{\mathop {\sum}\nolimits_{i = 1}^n {\frac{{O_i}}{{{{{{\mathrm{n}}}}}}}} }}$$

9 $${{{{{\mathrm{R}}}}}}^2 = \left\{ {\frac{{\mathop {\sum}\nolimits_{i = 1}^n {\left( {{{{{{\mathrm{O}}}}}}_{{{{{\mathrm{i}}}}}} - {{{{{\mathrm{O}}}}}}_{{{{{{\mathrm{ave}}}}}}}} \right) \times \left( {{{{{{\mathrm{P}}}}}}_{{{{{\mathrm{i}}}}}} - {{{{{\mathrm{O}}}}}}_{{{{{{\mathrm{ave}}}}}}}} \right)} }}{{\left[ {\mathop {\sum}\nolimits_{i = 1}^n {\left( {\left( {{{{{{\mathrm{O}}}}}}_{{{{{\mathrm{i}}}}}} - {{{{{\mathrm{O}}}}}}_{{{{{{\mathrm{ave}}}}}}}} \right)} \right)^2} } \right]^{0.5} \times \left[ {\mathop {\sum}\nolimits_{i = 1}^n {\left( {{{{{{\mathrm{P}}}}}}_{{{{{\mathrm{i}}}}}} - {{{{{\mathrm{P}}}}}}_{{{{{{\mathrm{ave}}}}}}}} \right)^2} } \right]^{0.5}}}} \right\}^2$$

10 $${{{{{\mathrm{Bias}}}}}} = \frac{{\mathop {\sum}\nolimits_{i = 1}^n {{{{{{\mathrm{P}}}}}}_{{{{{\mathrm{i}}}}}}} - \mathop {\sum}\nolimits_{i = 1}^n {{{{{{\mathrm{O}}}}}}_{{{{{\mathrm{i}}}}}}} }}{{\mathop {\sum}\nolimits_{i = 1}^n {{{{{{\mathrm{O}}}}}}_{{{{{\mathrm{i}}}}}}} }} \times 100{{{{{\mathrm{\% }}}}}}$$

11 $${{{{{\mathrm{NSE}}}}}} = 1 - \frac{{\mathop {\sum}\nolimits_{ - = 1}^n {\left( {P_i - O_i} \right)^2} }}{{\mathop {\sum}\nolimits_{ - = 1}^n {\left( {P_i - O_{Ave}} \right)^2} }}$$

where, O_i_ – Observed data, P_i_ – predicted data, O_ave_ – the average of the observed, P_ave_ – the average of the predicted, and n – sample count.

#### Water yield (WY) coefficient

The WY coefficient represents water availability in the various categories of land use. For each form of LUC, it can be determined using Eq. 12 (Li et al. 2018). The WY coefficient also shows the water yield conversion resulting from precipitation due to the effects of infiltration, saturation, and evapotranspiration (Singh et al. 2011). Climate variations were taken into account when determining the coefficient of water yield for each type of LUC.

12 $${{{{{\mathrm{Water}}}}}}\,{{{{{\mathrm{yield}}}}}}\,\left( {{{{{{\mathrm{WY}}}}}}} \right)\,{{{{{\mathrm{coefficient}}}}}} = \frac{{{{{{{\mathrm{WY}}}}}}}}{{{{{{{\mathrm{precipitation}}}}}}}}$$

#### LUC and climate variability effect on water provision

The main drivers for the change in water yield could be climate variability and LUC change. However, specific scenarios were considered to determine which one affected the change in water yield in the Winike watershed more. To this end, a scenario (1) without climate variability and a scenario (2) without LUC change were input into the InVEST water yield model. Under scenario (1), only LUC change was considered. Therefore, LUC data for 1988, 1998, 2008, and 2018 were provided as the input for the respective years. However, meteorological data were not recognized in the model in Scenario (1). In scenario (2), the raster reference evapotranspiration (ET_0_) and precipitation for 1988, 1998, 2008, and 2018 were provided as input. LUC data were not considered in the model in Scenario (2). The remaining model input parameters such as biophysical CSV table, season factor (Z), and water demand table were constant in the two scenarios. The two scenarios were run independently for each year using the InVEST water yield model based on the provided data.

## Results and Discussion

### Land Use/Cover Change

**Fig. 4:**
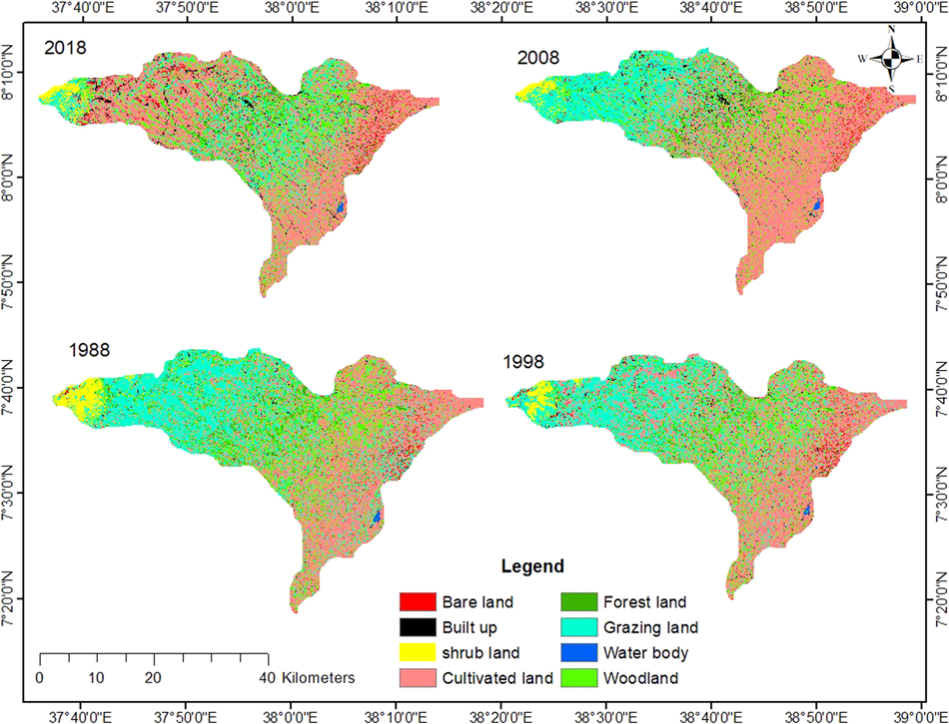
Maps of Land use and land cover changes for the reference years

The LUC analysis for 1988, 1998, 2008, and 2018 shows a massive conversion of LUC (Fig. 4). For example, forest and grazing land declined by 35.56% and 49.12%, respectively, whereas cultivated land grew by 33.01% (Table 2), which is indicative of a large-scale conversion of grazing and forest area into cultivated land in the last 30 years. Moreover, built-up and bare land increased by 109.58% and 65.19%, respectively. The major reason for the land dynamics in the investigated area could be an increase in population pressure, which led to the expansion of agricultural land through clearance of vegetated land and continuous ploughing of the existing agricultural land without fallowing and other conservation practices. Woodland also increased by 4.25% because of an expanding Eucalyptus plantation. It is of significant economic value to local farmers, greater than other crops. The change of grazing land and forest land into cultivated fields amounted to 19% and 1.84%, respectively, while the conversion of cultivated land into forest land was insignificant. Nevertheless, cultivated land was converted into grazing land by 7.07%. Overall, 45.89% of the land has never changed; for example, 31.79 thousand hectares (29%) of cultivated land have not changed for the last 30 years.

**Table 2:** The land use/cover changes per hectare in the last 30 years

LULC	1988	1998	2008	2018	Change (ha)	Change (%)
Bare land	1517.49	1612.26	1804.59	2506.68	989.19	65.19
Built-up land	1891.23	1427.22	2744.28	3963.6	3377.43	109.58
Shrubland	3245.22	2324.61	1816.02	2030.04	–1215.18	−37.45
Cultivated land	44954.5	51892.2	57442.5	59792.9	14838.4	33.01
Forests	7353.72	5209.74	4466.79	4738.86	–2614.86	−35.56
Grazing land	32876.3	30243	23267.3	16728.3	–16148	−49.12
Water bodies	252.18	254.34	280.98	242.01	–10.17	−4.03
Woodland	18397	16219.2	17360.1	19180.1	783.1	4.26

### Water Yield Change in the Watershed

**Fig. 5:**
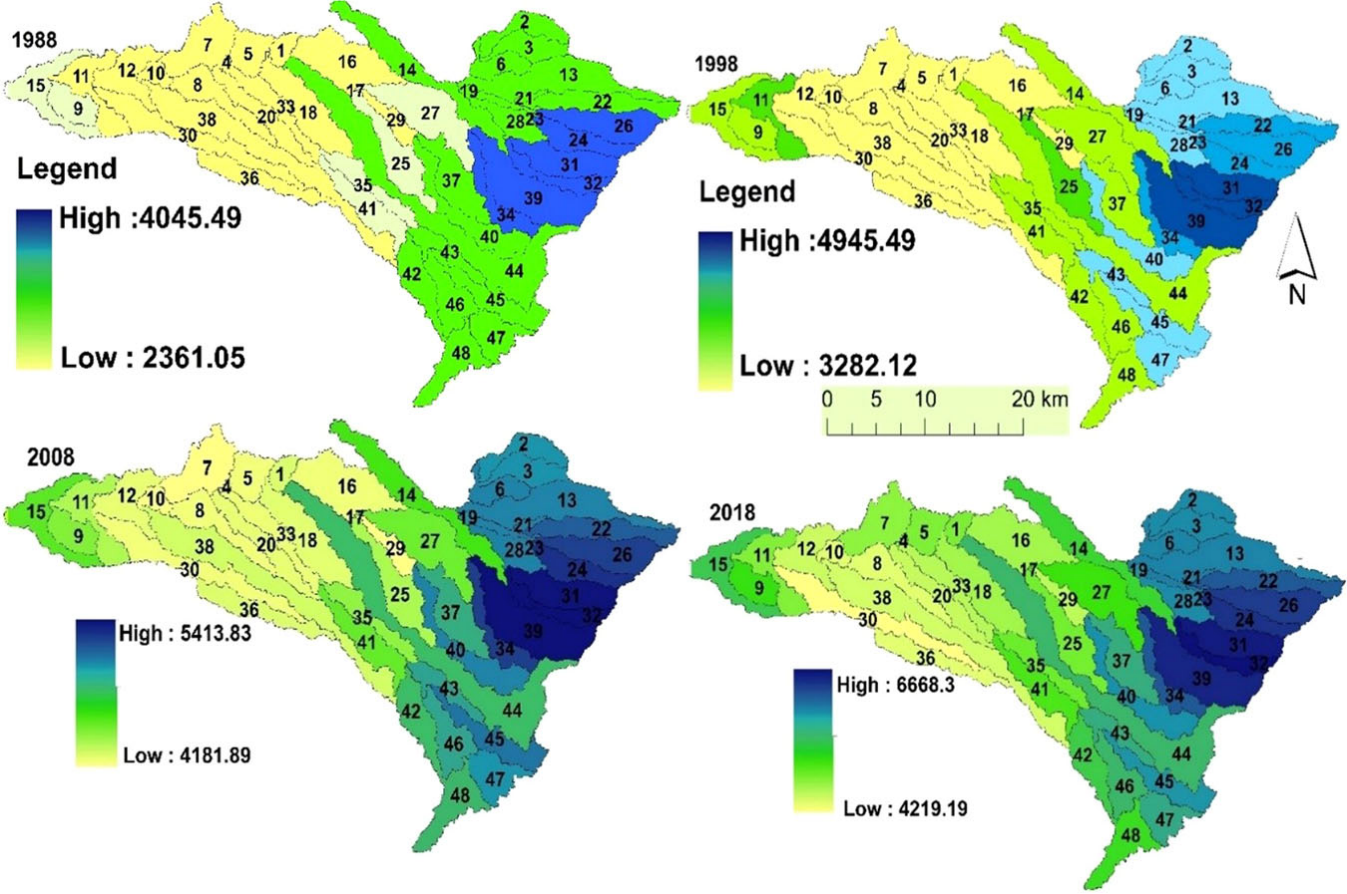
Spatial distribution of the mean water yield (m^3^/ha/year) for sub-watersheds

The spatial water yield was evaluated for different years, 1988, 1998, 2008, and 2018 (Fig. 5). The total annual water provision increased from 1.83 × 10^9^ m^3^ in 1988 to 3.35 × 10^9^ m^3^ in 2018, which is an increase of 83.21% in the last 30 years. In 1988, the water yield ranged from 2362 m^3^/ha to 4045 m^3^/ha, with an average below 2800 m^3^/ha. In 2018, the water yield was 4219 m^3^/ha to 6668 m^3^/ha, with a mean of 5130 m^3^/ha. Spatially, the greatest water provision in the last three decades was found in the eastern part of the watershed.

We recognize that there is a significant difference in the water yield model in every sub-watershed (SW) because of the differences in the climatic scenarios for the area. Sub-watersheds 31, 32, and 39 in the eastern part of the watershed contributed more water than the other sub-watersheds, with a mean value of 6589.32 m^3^/ha/year, 6670.56 m^3^/ha/year, and 6579 m^3^/ha/year, respectively. Sub-watersheds 8, 29, and 30 provided the smallest amounts of water in each reference year for the last three decades. The higher-altitude area (eastern part of the watershed) also encompassing SW24, SW26, SW31, SW32, and SW39 had higher precipitation and lower temperatures (meteorological data analysis around these SWs recorded lower temperatures), and the water yield is correspondingly higher there. Moreover, precipitation was relatively high (1528 mm), and AET and PET were low (578 mm and 589, respectively) in SW32, while in sub-watershed 30 precipitation was the lowest (1366.5 mm) and AET and PET were 660 mm and 678 mm, respectively. Therefore, the differences in precipitation, incoming solar radiation, and temperature might cause significant changes in the water yield in the landscape. These conclusions are consistent with similar research findings by Yang et al. (2020b) in northwest China.

Water yield conversion from one sub-watershed to another was noted from 1988 to 2018. The average water yield in each sub-watershed from 1988 to 2018 indicated a constant and increasing trend. For example, water yield in SW1, SW4, SW5, SW12, and SW16 was lower between 1988 and 2008, but it started an increasing trend in 2018 while in SW8, SW29 and SW30, it was constantly the lowest (Fig. 5).

### Effect of Land Use/Cover Change on Water Yield

The WY coefficients of the investigated LUC types exhibited substantial differences (*p* < 0.05), and water provision was prone to variations due to LUC change (Kocur-Bera 2018; Sharp et al. 2018). Therefore, the analysis demonstrated a significant variation in the water yield in each LUC type (Table 3). The rainfall-runoff characteristics, PET and AET of a basin can be modified by LUC change. It consequently affects the hydrological parameters of a watershed (Defersha and Melesse 2012). These can be a change of evapotranspiration, infiltration, water retention, and water availability (Sánchez-Canales et al. 2012). Based on the climate data for the four reference years, the simulated average AET and PET for different LUC types showed significant differences (*p* < 0.05).

**Table 3:** Water yield, actual and potential evapotranspiration from 1988 to 2018 in each LUC class

Year		Shrubland	Cultivated land	Forest land	Grazing land	Woodland	Built-up land	Bare land	Mean
1988	WY	3078	2934	3883	2587	2518	2200	2400	2800
	AET	792	449	794	505	595	310	325	510
	PET	799	553	607	511	590	314	336	501
1998	WY	2854	2798	4500	2754	2967	2987	3154	3145
	AET	804	556	815	598	510	412	432	547
	PET	808	560	732	572	514	420	447	536
2008	WY	2812	2754	5646	3040	3051	4200	4434	3705
	AET	887	565	843	597	528	345	368	548
	PET	896	569	757	580	539	354	378	539
2018	WY	2885	2867	6324.9	5484.9	4864.9	6764.9	5964.9	5130
	AET	903	657	921	601	712	356	389	620
	PET	917	662	929	609	720	367	397	628
Change WY		−193	−67	2442	2898	2347	4565	3565	2330
Change AET		213	208	127	96	117	46	64	110
Change PET		211	109	322	98	130	53	61	126

WY: Water yield (m^3^/ha), AET: Mean actual evapotranspiration (mm), PET: Potential evapotranspiration (mm)

The LUC change effects on water yield were not the same because the change of land use causes a change in soil properties and biodiversity, and then the underlying surface water, which affects runoff. This concentration process causes changes in the water cycle in the watershed, eventually affecting water yield (Lang et al. 2017).

This study shows that built-up and bare land water yield increased by 206.96% and 148.54%, respectively. This could be due to the change in vegetation and conversion of land use into built-up and bare land. The increase in water yield intensity in the built-up and bare land was due to impervious surfaces deteriorating the infiltration and concentration time (Liu et al. 2013). Built-up land covered only 3.6% of the total area but accounted for around 42% of the total water provision in 2018 due to its high-water yield coefficient value (5).

However, the AET and PET were negatively correlated with the increase in built-up land. This contributes to more water yield in built-up land than in forest land. A similar report also shows that built-up land generates more water yield while land with vegetation cover triggers lower water yield (Im et al. 2009). Similar studies by Yang et al. (2013) and Zhao et al. (2017) show an increase of flooding in urban areas and that land not covered by vegetation cannot hold water in the soil for long. The analysis also shows that the water yield coefficient of built-up land and bare land was higher than for vegetated land, which indicates the absence of canopy that contributes to the lack of water infiltration, low evapotranspiration, and soil water retention (Arunyawat and Shrestha 2016; Jujnovsky et al. 2017).

Forest cover declined by 35.56% over the last three decades. However, the water yield in forests increased by 3042 m^3^ (63%) between 1988 and 2018, which is still not as much as for built-up and bare land, because the forest has deep root systems and high permeability that facilitates storage of water as soil moisture in the pores of the soil and through interception by leaves. Due to the shade services of the forest, excessive sunlight that would cause more water loss in the underlying soil is also low. Therefore, the dissipation of water by evaporation in forest land is lower even if transpiration is higher. The water yield coefficient for forest land is also higher (3.8) compared with other LUC types because of the high infiltration, water-holding, and groundwater recharge capacity of forest land. This is supported by Li et al. (2018), who demonstrated that water yield increased after afforestation.

Cultivated land shows a low capability of interception and shallow root systems, and consumes a huge rate of water for the growth of crops, which leads to a substantial loss of water (Yang et al. 2020b). As a result, water provision in cultivated land was lower (2885 m^3^/ha) as compared to forest land (6925 m^3^/ha) in 2018, which leads to the record lowest water yield coefficient (1.5) as compared to other land uses types due to low saturation and infiltration (no interception) and significant water loss in cultivated land. Although the cultivated land area increased by 14,838.4 ha (33%), its water provision declined by 67 m^3^/ha (2.28%).

Most of the shrubland area was located in the Omo Gibe valley, which is a hot environment. Its area has declined dramatically due to agricultural expansion. As a result, the highest AET (903 mm) and PET (917 mm) were estimated in the model for shrubland. It furthermore led to the decline in water provision in shrubland by 6.27%, from 3078 m^3^/ha in 1988 to 2885 m^3^/ha in 2018. Grazing land also declined by 49.12%, whereas its water provision increased by 112%. This might be due to significant infiltration in grazing land. Woodland area also increased by 4.25% in the last 30 years, and its water yield also increased by 23% (Table 4).

**Table 4:** Comparison of water yield (WY) (m^3^) caused by climate variability and LUC change

Year	WY for LUC-only scenario (10^9^)	WY for climate change-only scenario (10^9^)	Water yield with actual scenarios (10^9^)
1988	1.72	1.72	1.72
1998	1.74	1.87	1.92
2008	1.76	2.09	2.14
2018	1.99	2.31	3.35

**Fig. 6:**
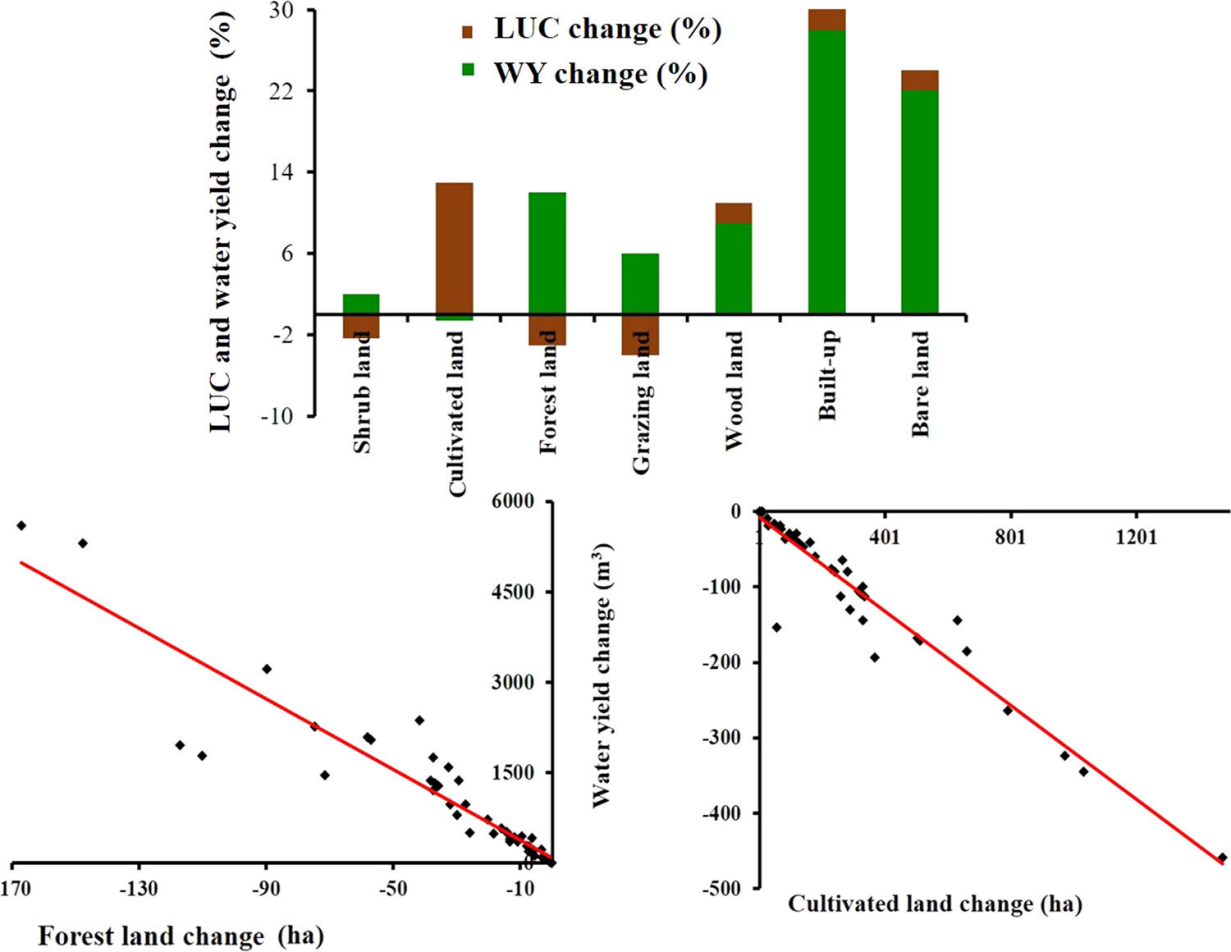
Correlation of WY with LUC changes

Each LUC change has demonstrated a correlation with a water yield change (Fig. 6). The Pearson correlation investigation showed that the forest land change was negatively correlated with the water yield change (Fig. 6a) (*r* = –0.78, *p* < 0.05). The change in cultivated land was negatively correlated (Fig. 6b) with the change in water yield (*r* = –0.67, *p* < 0.05). Moreover, the change in water yield was positively correlated with the change in woodland. Grazing land and shrubland changes were negatively correlated with the water yield change. Built-up and bare land showed positive correlations with water yield changes (*r* = 0.82, *p* < 0.05 for built-up and *r* = 0.73, *p* < 0.05 for bare land).

### Climate Variability and LUC Change Effect on Water Provision

The analysis showed that climate variability had more influence on water yield than LUC change (Table 5). The scenario without LUC changes (only climatic data analysis), substantially increased water yield in the Winike watershed by 68%. This shows that the main factor for the increased water yield is climate variability. Furthermore, we observed a strong impact of precipitation on water yield in the watershed. The scenario that investigated the effect of LUC change (without climate data) revealed that water yield increased by 31%, but it was not significant compared to the scenario for climate data. A similar study conducted by Dai et al. (2020), shows that water yield contributed by land use and climate variability increased by 26.94% and 73.06%, respectively. Therefore, the study shows that climate variability was the main driving force for the spatial and temporal changes in water yield in the study area.

**Table 5:** The contribution of water from the watershed to the dams

	Total water yield	Water consumption			Realized supply model
		Industry	People	Animals	
Amount (m^3^)	3 × 10^9^	5 × 10^8^	4 × 10^6^	2 × 10^7^	2.81 × 10^9^
Percent (%)		13.33	0.11	0.45	72.23

### Watershed’s Water Yield Contribution

The Winike watershed contributes a substantial water yield to the hydropower generation found in the downstream part of the watershed even if exploited by human activities and affected by LUC change and climate variability. The average daily water consumption by a household was 79.77 liters (0.080 m^3^) (Table S3). The total annual water consumption by the human population in the watershed was 4.2 × 10^6^ m^3^ (38.29 m^3^/ha/year) (Table 5).

The total daily and annual water consumption by livestock in the watershed was 48,250 m^3,^ and 2 × 10^7^ m^3^, respectively (Table S4), and the annual water extraction by the industry was 5.2 × 10^8^ m^3^. This is a grave threat to the surrounding ecosystem and the downstream hydroelectric power plants by limiting the water delivery to the dams in the watershed.

The actual InVEST water yield model generated 3.35 × 10^9^ m^3^ of water yield in the watershed (Table 4). The total consumed water by industry, people, and animals amounted to 5.4 × 10^8^ m^3^. The analysis shows that the major consumers of water were the industry (13.33%), followed by animals (0.45%), and people (0.11%). Therefore, the watershed water yield contribution to the hydropower generation was 2.8 × 10^9^ m^3^ (72.22%) (Table 5). This indicates that the watershed has a vast water potential and can contribute to the downstream ecosystem conservation, but the climate change and extra exploitation of water by the business and human activities call for careful handling of the watershed.

### Model Sensitivity and Validation

The analysis shows that the quantification of water yield is significantly sensitive to input climate data (precipitation and reference evapotranspiration). A 10% increase in precipitation generated an increase in water yield by 66%, from 3345 m^3^/ha to 5566 m^3^/ha. Similarly, a 10% decline in precipitation generated a drop of 48%. When ET_0_ increased by 10%, water yield decreased by 18%, and when ET_0_ decreased by 10%, water yield increased by 7.6%. This demonstrates that although P and ET_0_ had a significant impact on changes in water yield, precipitation had an even greater influence than ET_0_.

For a 10% increase in datasets of *Kc*, AWC, and root depth, the model water yield decreased 0 to 2%. As these input variables decreased by 10%, water yield increased by 0 to 3%. The analysis shows that the sensitivity of water yield to changes in *Kc*, AWC, and root depth was insignificant. When the *Z* factor (season factor) value changed from the baseline of 29–32, water yield decreased by 12%. When the *Z* value changed from the baseline of 29 to 0, the water yield increased by 60%. A change in the *Z* factor from the baseline value of 29 to a value of 12, increased the water yield by approximately 5.5%. This shows that an increase in the value of the Z factor caused water yield to decrease.

Generally, the watershed water yield analyzed with the model is highly sensitive to the variation in values of some model input parameters and other model input parameters do not contribute to the variation in water yield to a large extent. In other words, for less decisive parameters, the model shows a little variation in the water yield due to their little impact.

The predicted water yield was consistent with the corresponding estimates from observed data (*R*^*2*^ = 0.91, RRSME = 0.92, NSE = 0.85, *P* < 0.01) (Table 6). The mean value of the observed and simulated water yield was 4687.75 m^3^/ha and 4494.47 m^3^/ha, respectively, with a difference of 192.28 m^3^/ha. The InVEST model could predict water yield with a low discrepancy, an error of –2.1% (underestimation), suggesting that the LUC effects and its parameters were sufficiently recognized by the model for predicting the water yield in the study watershed. Therefore, a ± 10% difference in the accuracy of the model is considered to be a very good rating for sensitivity analysis of this study, as suggested by Moriasi et al. (2007).

**Table 6:** Validation of the InVEST water yield model using the observed data

Years	Stations	Observed	Predicted	PBIAS^a^	R^2^	RRSME^a^	NSE^a^
1988	Agena	3483.59	3292.61	−1.04	0.82	0.6	0.75
	Imdiber	3309.41	3207.98	−0.55	0.85	0.77	0.92
	Gunchre	3916.15	4013.79	−0.3	0.7	0.84	0.98
	Gumer	4069.62	3427.12	−0.49	0.77	0.63	0.57
	Merbe azernet	3942.64	3879.2	−0.35	0.87	0.8	0.96
1998	Agena	3976.6	3876.82	−0.54	0.85	0.63	0.9
	Imdiber	3976.6	4075.58	−0.92	0.7	0.99	0.89
	Gunchre	3988.22	3885.58	−0.56	0.77	0.64	0.9
	Gumer	4244.03	3809.58	−0.91	0.87	0.65	0.74
	Merbe azernet	4011.48	3917.99	−0.51	0.77	0.88	0.83
2008	Agena	4623.08	4500.47	−0.67	0.65	0.76	0.88
	Imdiber	4677.35	4477.35	−1.09	0.72	0.87	0.95
	Gunchre	5219.96	5019.96	−1.09	0.82	0.96	0.85
	Gumer	4818.43	4585.87	−1.09	0.82	0.94	0.96
	Merbe azernet	4785.87	4707.53	−0.61	0.85	0.75	0.89
2018	Agena	5968.77	5668.77	−1.64	0.77	0.93	0.91
	Imdiber	5860.25	5560.25	−1.9	0.87	0.89	0.87
	Gunchre	6185.82	5885.82	−2.3	0.9	0.92	0.98
	Gumer	6402.86	6102.86	−2.6	0.85	0.97	0.91
	Merbe azernet	6294.34	5994.34	−2.74	0.87	0.91	0.95
	Average	4687.75	4494.47	−1.10	0.80	0.82	0.88

^a^ RRMSE = Residual Root Mean Square Error; NSE = Nash-Sutcliffe Efficiency; and PBIAS = Percentage Bias Error

### Policy Implication

Water yield in individual LUC types could contribute to consumptive water for the industry, people, animals, irrigation, and hydroelectric power generation (Brauman et al. 2007). There is also a probability of increasing flood (Rutkowska et al. 2017), causing erosion that affects downstream ecosystem services such as dams (Halecki et al. 2018). The reduction of excessive runoff is the main challenge for land-use planning and agricultural water resource management. This study recommends increasing forest land and reversing the conversion of vegetated land into built-up land and bare land because these land-use types could hinder permeability and maximize evaporation. Moreover, precipitation can increase in forest land because of micro-climatic interactions (Brauman et al. 2012). Many research findings also show that a decline in forest land results in an increase in flood peaks, annual flow, and flood volume (Kurowska et al. 2020; Rogatka et al. 2021; Romagnoli et al. 2018). Therefore, urgent conservation measures are required in the watershed to mitigate water yield stress caused by LUC and climate change and its effects. It is also essential to improve green policies such as conservation of forests or tree planting and implement Soil Water Conservation (SWC) to maintain water retention and maximize regulating capacities of ecosystems (Xu et al. 2017). This can be achieved by mass stakeholder’s participation, maximization of indigenous ecological knowledge, implementation and improvement of environmental policy/law and boosting the awareness of the local community (Chodkowska-Miszczuk et al. 2021).

## Conclusions

Omo gibe has a huge water resources potential, which leads to the construction of numerous hydropower dams in the lower parts of the basin and contributes to the improvement of the livelihood of the local community. However, many research finding shows that the dams and the associated ecosystem services will be damaged if appropriate conservation measurements are not implemented in the higher-altitude part of the basin or watersheds. The current challenges are catastrophic factors in the higher-altitude part of the basin, the Winike watershed, such as high population pressure, leading to the expansion of agriculture and removal of vegetation cover in search for traditional fuelwood, which resulted in deforestation and biodiversity loss. Therefore, the stakeholders responsible for managing and planning water resources should consider reducing the vegetation cover decline, introducing sustainable grazing, restoring and protecting water bodies, maximizing regreening, and reducing land degradation to maintain water resources in the basin.

The present research analyzed spatiotemporal changes in water yield in the Winike watershed from 1988 to 2018. Quantifying and mapping water provision is useful for the sustainable management of water resources and other related ecosystem services. In this study, the water yield rate of each LUC was analyzed using the InVEST water yield model.

This research also shows the effect of land use/cover change and climate variability on water yield. It is more affected by climate variability as compared to land use/cover change. Moreover, the identification of factors that affect water yield more is important for the management of water resources. The analysis shows that precipitation had a more significant impact on the estimation of water yield compared to other climate factors.

This research also shows a spatial and temporal distribution of water yield by LUC type, which is helpful for saving time, energy and resources since it can be easily visualized on a map. It is important for the management of the land-use types to know the water yield potential of each land use/cover type, since some land-use types cause greater water loss.

The total annual water provision increased from 1.83 × 10^9^ m^3^ in 1988 to 3.35 × 10^9^ m^3^ in 2018, which is an increase of 83.21% in the last 30 years. Sub-watersheds 31, 32, and 39 had the highest water yield, while sub-watersheds 8, 29, and 30 had the lowest water yield. Built-up land had the highest water yield, followed by bare land and forest land. The InVEST model could predict water yield with a low error of –2.1%, suggesting that the LUC and climate factors are sufficiently recognized by the model. The InVEST water yield model estimation was consistent with the corresponding observed water yield from MOWIE data (*r* = 0.91, *P* < 0.05). The watershed’s water yield contributed to hydropower generation by 72%; the remaining water was withdrawn by the industry, livestock, and people. This indicates the future threats for the water resource in the watershed. The analysis of water yield in sub-watersheds contributes to the management of small water supply, flood control, as well as hydropower production in the watershed. Our findings could be used to maintain and manage water resources in the watershed by engaging stakeholders regarding the considerable reduction of forest cover, increase in cultivated land, and other important land-use type conversions.

For determination of water yield, we recommended that the InVEST model is preferred as compared to SWAT and others models because SWAT is more time-consuming and computer-intensive in terms of data processing. Moreover, the InVEST water yield model is easier to use with fewer input data, and yet its output is more explanatory, understandable and easily interpretable. In addition, the output of the InVEST model can be shown at a pixel level (small area), whereas other models, such as SWAT, show the output at a hydrological response unit (sub-watershed) level, which is indicative of low precision. Therefore, the introduction of the InVEST water yield model is helpful for processing hydrological information on a landscape, which is important to determine which parts of the watershed have more degraded water situation. It also shows what factors affect water yield more in a sensitivity analysis. Therefore, the InVEST model is more appropriate to show sensitivity analysis.

Different projects associated with water resources might be designed for irrigation, dam construction, fish pond, water business companies, industry etc. Knowing the spatial water potential is important for such projects, saving time, energy, and resources for the stakeholders. Therefore, this research contributed to the design of projects associated with water resources, which encourages water development research.

Nature-based solutions are an increasingly popular approach to water resource management. Therefore, producing hydrological information on a landscape to inform decision-makers based on appropriate data is important and can help implement as a pilot project based on appropriate data. Moreover, ecosystems are interconnected, so preservation of the Winike watershed through a scientifically-based watershed development plan will ensure sustainability in the downstream ecosystem.

The basin is a significant contributor to the Ethiopian green economy by generating hydropower. Therefore, locally-initiated research, such as on the water yield potential of the Winike watershed, will contribute to achieving suitable development goals. Different types of projects must be implemented primarily at the local level to reduce vulnerabilities and build resilient communities to eventually solve global challenges. Therefore, such types of research help develop important local projects, which has a significant impact at a national, regional, and global level.

Moreover, spatial assessment of water yield helps planners and decision-makers identify the priority areas for conservation. Additionally, quantitative and qualitative assessment of water yield plays an important role in the socio-economic development of human societies as well as ecosystem health. This will help ensure the sustainability of the basin ecosystems.

## Supplementary information

Supplementary Material

## Data Availability

Not applicable.
