# Supplementary material for: Evaluation of Water Provision Ecosystem Services Associated with Land Use/Cover and Climate Variability in the Winike Watershed, Omo Gibe Basin of Ethiopia
Source: Environ Manage. 2021 Dec 8;69(2):367–83. doi: 10.1007/s00267-021-01573-9 (PMC8789729; doi:10.1007/s00267-021-01573-9)
Supplement: Supplementary file 1 — Supplementary Material [file 267_2021_1573_MOESM1_ESM.docx]

**Table S1.** Biophysical table of the InVEST water yield model

| LUC_ desc | lucode | *Kc* | Root depth (mm) | LUC_ veg |
| --- | --- | --- | --- | --- |
| Bare land | 1 | 0.5 | 700 | 0 |
| Built-up land | 2 | 0.3 | 500 | 0 |
| Shrub land | 3 | 0.39 | 8,000 | 1 |
| Cultivated land | 4 | 0.65 | 5,500 | 0 |
| Forest land | 5 | 1 | 10,070 | 1 |
| Grazing land | 6 | 0.85 | 6,500 | 1 |
| Waterbody | 7 | 1 | 500 | 0 |
| Woodland | 8 | 1 | 8,500 | 1 |

**Table S2.** Data required for reference evapotranspiration (ET_0_) of the stations

| Stations | Parameters | 1988 | 1998 | 2008 | 2018 |
| --- | --- | --- | --- | --- | --- |
| Imdiber | RA | 137.52 | 140.75 | 136.03 | 137.17 |
|  | Max T | 26.01 | 26.5 | 27.3 | 27.7 |
|  | Min T | 12.34 | 12.94 | 13.34 | 13.8 |
|  | Precipitation | 846.7 | 1,096.7 | 1,230.5 | 1,366.5 |
|  | ET_0_ | 402.3 | 507.7 | 550.8 | 642.3 |
| Agena | RA | 138.21 | 138.76 | 139.81 | 139.9 |
|  | Max T | 25.3 | 25.7 | 26.13 | 26.23 |
|  | Min T | 13.14 | 13.23 | 13.31 | 13.42 |
|  | Precipitation | 1,139.6 | 1,063.4 | 1,230.5 | 1,466.5 |
|  | ET_0_ | 397.4 | 434.56 | 456.67 | 570.7 |
| Gunechre | RA | 132.45 | 132.89 | 133 | 133.45 |
|  | Max T | 25.2 | 25.6 | 25.7 | 26.45 |
|  | Min T | 12.14 | 12.34 | 12.9 | 13.01 |
|  | Precipitation | 1,143.2 | 1,190.7 | 1,329.5 | 1,439.5 |
|  | ET_0_ | 396.7 | 420.56 | 480.9 | 580.3 |
| Gumer | RA | 133.05 | 133.22 | 133.34 | 133.56 |
|  | Max T | 25.02 | 25.23 | 25.56 | 25.87 |
|  | Min T | 11.78 | 11.94 | 11.99 | 12.23 |
|  | Precipitation | 1,299.2 | 1,343.7 | 1,438.5 | 1,528.23 |
|  | ET_0_ | 391.8 | 429.9 | 460.43 | 565.8 |
| Gubre | RA | 131.15 | 131.34 | 131.45 | 131.95 |
|  | Max T | 26.11 | 26.45 | 26.89 | 27.75 |
|  | Min T | 12.24 | 12.87 | 13.21 | 13.9 |
|  | Precipitation | 1,023.2 | 1,131.4 | 1,270.7 | 1,345.2 |
|  | ET_0_ | 410.34 | 495.6 | 545.78 | 632.32 |
| Merab azerent | RA | 137.01 | 137.87 | 138.13 | 139.1 |
|  | Max T | 25.09 | 25.67 | 26.03 | 26.21 |
|  | Min T | 12.54 | 13.01 | 13.51 | 13.67 |
|  | Precipitation | 892.23 | 1,120.00 | 1,235.90 | 1,267.34 |
|  | ET_0_ | 390.70 | 411.90 | 460.90 | 550.12 |
| Average | RA | 134.90 | 135.81 | 135.29 | 135.86 |
|  | Max T | 25.46 | 25.86 | 26.27 | 26.70 |
|  | Min T | 12.36 | 12.72 | 13.04 | 13.34 |
|  | Precipitation | 1,057.36 | 1,157.65 | 1,289.27 | 1,418.88 |
|  | ET_0_ | 398.21 | 450.04 | 492.58 | 590.26 |

**Table S3.** The population and average water consumption in the watershed

| Districts (*woreda*) | Population | Daily consumption (m^3^/day) | Average people per household | HH water consumption (m^3^/day) | Total water consumption (m^3^) | Percent |
| --- | --- | --- | --- | --- | --- | --- |
| Ezha | 155,851 | 1,767.35 | 7 | 0.079 | 636,246.12 | 15.13 |
| Gumer | 173,806 | 1,623.348 | 9 | 0.084 | 584,405.29 | 13.9 |
| Geta | 112,340 | 1,096.438 | 8 | 0.078 | 394,717.82 | 9.39 |
| Cheha | 218,687 | 2,593.628 | 7 | 0.083 | 933,706.02 | 22.21 |
| Enemor Ener | 248,458 | 3,065.972 | 6 | 0.074 | 1,103,749.82 | 26.25 |
| Merab Azernet | 153,055 | 1,532.081 | 8 | 0.08 | 551,549 | 13.12 |
| Average | 1,062,197 | 1,946.47 |  | 0.08 | 4,204,374.07 | 100 |

**Table S4.** Populations of livestock and annual water consumption in cubic meters (m^3^) per individual livestock type in the watershed

|  | Districts | | | | | | Total daily | Annual |
| --- | --- | --- | --- | --- | --- | --- | --- | --- |
| Animal types | Eza | Gumer | Cheha | Enemor | Merab | Geta |  |  |
| Cows | 63,973 | 69,446 | 112,737 | 434,229 | 85,329 | 74,483 | 1,735,316 | 1*10^7^ |
| Daily water requirement ^a^ | 2,878.785 | 3,125.07 | 5,073.17 | 19,540.3 | 3,839.81 | 3,351.74 | 37,808.865 |  |
| Sheep and goats | 63,776 | 78,145 | 25,900 | 240,849 | 55,180 | 43,583 | 1,243,371 | 2*10^6^ |
| Daily water requirement ^b^ | 701.536 | 859.595 | 284.9 | 2,649.33 | 606.98 | 479.413 | 5,581.763 |  |
| Horses/donkeys/mules | 20,382 | 27,573 | 10,987 | 27,740 | 12,307 | 8,995 | 250,284 | 2*10^6^ |
| Daily water requirement ^c^ | 917.19 | 1,240.79 | 494.415 | 1,248.3 | 553.815 | 404.775 | 4,859.28 |  |
| Total livestock water requirement (m^3^) | | | | | | | 48,250 | 2*10^7^ |

*a, b, c = cows; sheep and goats; horses/donkeys/mules, respectively*
